# Supplementary material for: A universal method for in situ control of stoichiometry and termination of epitaxial perovskite films
Source: Nat Commun. 2025 Sep 29;16:8587. doi: 10.1038/s41467-025-63608-7 (PMC12479741; doi:10.1038/s41467-025-63608-7)
Supplement: Supplementary file 1 — Supplementary Information [file 41467_2025_63608_MOESM1_ESM.pdf]

SUPPLEMENTAL INFORMATION

for

A universal method for *in situ* control of stoichiometry and termination of perovskite films

B. A. Davidson[1,2*], A. Yu. Petrov[2], F. Li[1], R. Pons[3], P. Sosa-Lizama[3], H. Shin[1,4], C. Liu[1], P. Parisse[2], P. Torelli[2], G. Cristiani[3], Y. Eren Suyolcu[3], P. A. van Aken[3], G. Logvenov[3], G. Kim[3], X. X. Xi[5], E. Benckiser[3*] and K. Zou[1,4]

1 Quantum Matter Institute, Univ. of British Columbia, 2355 East Mall, Vancouver BC V6T 1Z4

2 CNR/IOM-TASC, Area Science Park-Basovizza, Trieste Italy 34149

3 Max Planck Institute for Solid State Research, Heisenbergstrasse 1, 70569 Stuttgart, Germany

4 Dept. of Physics and Astronomy, Univ. of British Columbia, 6224 Agricultural Road, Vancouver BC V6T 1Z1

5 Temple University, Dept. of Physics, Philadelphia PA 19100

* email: bruce.davidson@ubc.ca and benckise@fkf.mpg.de

This Supplemental Information contains:

Figure 1: RHEED geometry and ROI analysis (pg. 2)

Figure 2: $LaFeO_3$ RHEED diffracted beam rocking curves (pg. 3)

Note 1: A general method to calibrate stoichiometry and fluxes *in situ* using RHEED (pg. 4)

Figure 3: RHEED intensity versus time during $(Sr_{0.25}Eu_{0.25}La_{0.25}Nd_{0.25})TiO_3$ flux calibration by shutter method (pg. 6)

Figure 4: XRD on 50 u.c. HEO $(Sr_{0.25}Eu_{0.25}La_{0.25}Nd_{0.25})TiO_3$ film on STO (pg. 10)

Figure 5: XRD on STO films grown on different substrates (pg. 11)

Figure 6: XRD on STO films grown with Sr_n / Ti_n cycles, $1 < n < 5$ (pg. 12)

Figure 7: RBS spectra of perovskites grown with shutter method (pg. 13)

Figure 8: Diffracted "double peak" oscillations in Ti^{4+} perovskites (pg. 14)

Figure 9: "Double peak" oscillations in mixed-valence manganites (pg. 15)

Figure 10: "Double peak" oscillations in ferrites (pg. 16)

Figure 11: "Double peak" oscillations in $LaNiO_3$ and $LaAlO_3$ (pg. 16)

Figure 12: RHEED "double peak" oscillations during PLD growth (pg. 17)

Figure 13: RHEED diffracted oscillations vs incidence angle – (100) azimuth (pg. 18)

Figure 14: RHEED diffracted oscillations & rocking curves – (110) azimuth (pg. 19)

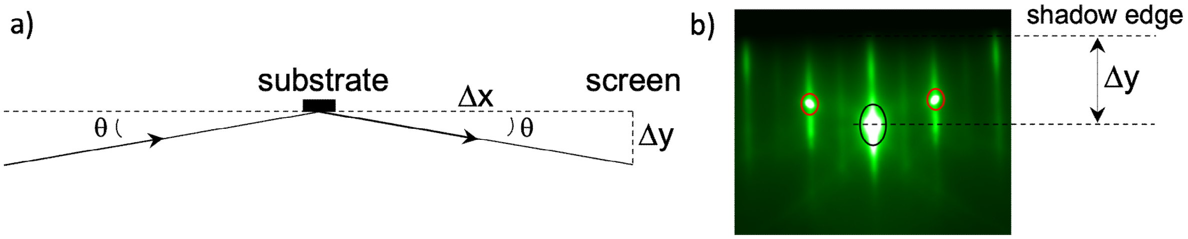

Supplementary Figure 1: RHEED geometry and ROI analysis

Calibration of the incidence angle under ideal conditions: the incidence angle is given by tan(Δy/Δx), where Δx is the distance from the sample center to the RHEED screen (given by the chamber dimensions) and Δy is measured directly on the phosphor screen. In our system (RHEED-15 hardware with kSA-400 software, kSpace, Inc., Ann Arbor MI), the incidence angle can be changed continuously by varying two coils for the electron beam ("deflection" and "rocking") that control the angle while allow to maintain the beam on the sample center. Fitting these deflection coil voltages with e.g. a 5th-order polynomial allows the kSA-400 software to adjust the incidence angle from 0.5-5 degrees in our chamber via a DAC board in the PC. The software allows definition of regions of interest (ROIs) in the image (red and black ellipses) and can calculate and plot the average intensity within each ROI in realtime during acquisition. The software permits the ROI to track the spot centroids if they move between sequential frames. This allows the acquisition of a rocking curve: a movie is acquired as the beam is scanned, the ROIs track the spot positions and their intensities are plotted in real time. In the geometry described in the main text, during shuttered film growth of $SrTiO_3$ the amplitude of the diffracted intensity oscillations during growth cycles can be adjusted to ~50% of full scale for our 14-bit CCD by fixing the RHEED filament emission current at 0.1 μA and ellipse dimensions of 80 by 150 pixels, for typical conditions of 10 keV and $P_{O2} = 1x10^{-6}$ torr.

In any deposition chamber, ideal conditions are never satisfied primarily because of residual magnetic fields that deviate the beam trajectory from a straight line. Thus, incidence angle calibrations will vary for different chambers, and this should be kept in mind when comparing experimental data between chambers or with calculated rocking curves. Nonetheless, as described in the main text, knowledge of the absolute incidence angle is not necessary to perform flux calibration using the method of Figs. 2 and 3 of the main text, as long as the incidence angle can be adjusted to satisfy the specific diffraction condition associated with the maximum intensity oscillations of the diffracted intensities during shuttered cycles.

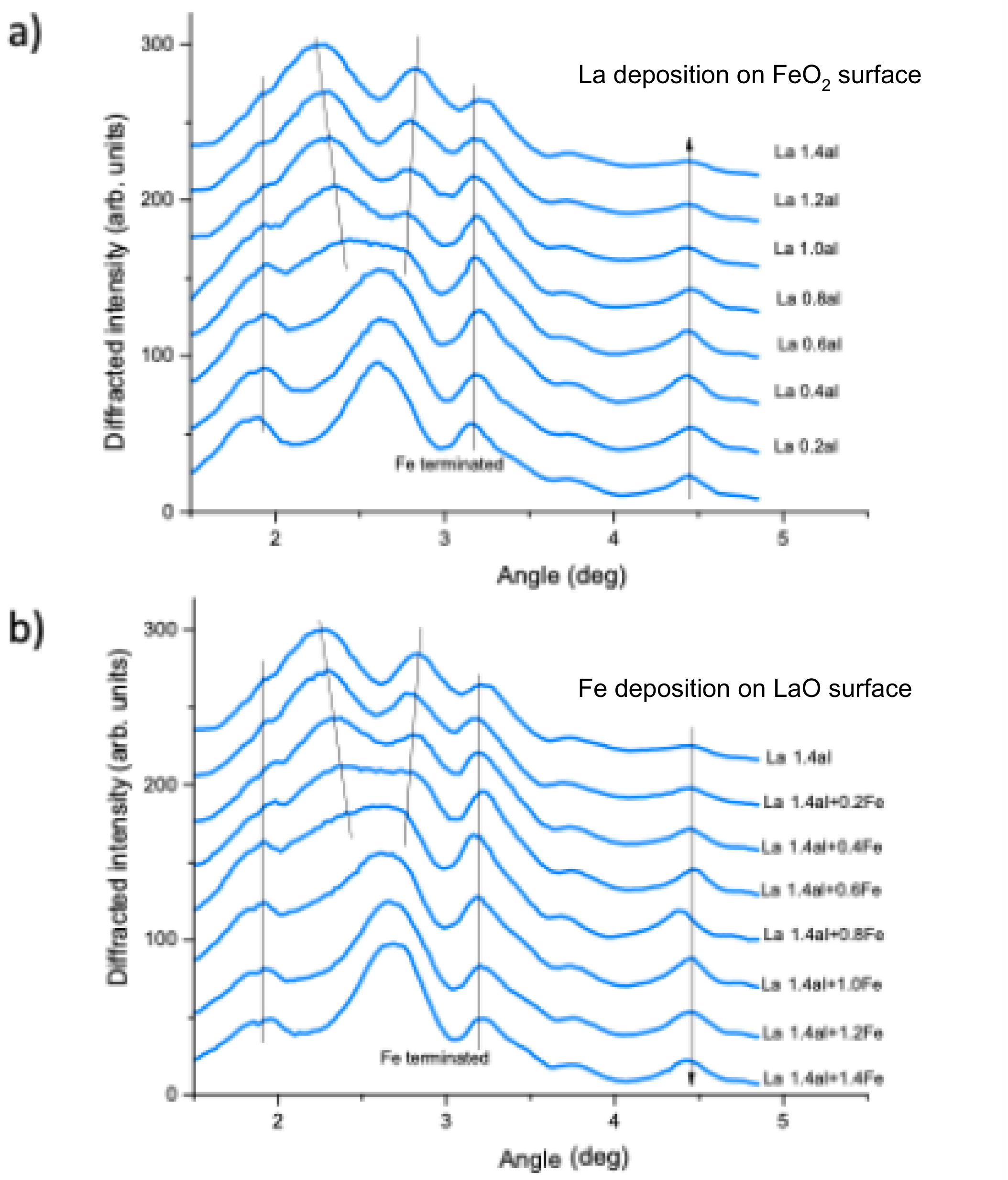

Supplementary Figure 2: RHEED diffracted-beam rocking curves during interrupted "alternating-shutter" growth of $LaFeO_3$

(a) Rocking curves of the diffracted intensity during La deposition starting close to FeO_2 termination. Curves are measured during growth interruptions after incremental deposition of 0.2 La layers, repeated to a total of 1.4 La layers; intensities are normalized to the maximum of each scan. Grey lines are a guide for the eye indicating shifts in the peak positions, and curves are shifted for clarity.

(b) Rocking curves during the return to FeO_2 termination by incremental deposition of 0.2 Fe layers. Note the similarity of rocking curves for the same net La coverage (e.g. comparing "La +0.6" and "La +1.4 followed by Fe +0.8"). Growth conditions: P_{O2}=2x10^{-6} mBar, $T_{substrate}$=780°C.

Supplemental Note 1: A general method for *in situ* calibration of stoichiometry and fluxes during perovskite growth using RHEED

Figure 3 in the main text describes an approach to calibrate perovskite ABO_3 growth entirely using *in situ* RHEED methods by combining shuttered growth with codeposition. Here we outline the method in more detail using $SrTiO_3$ growth as an example, and then extend the method to calibrate multiple A-site fluxes to grow the high-entropy phase $Sr_{0.25}La_{0.25}Eu_{0.25}Nd_{0.25}TiO_3$.

Prior to growth, the Sr and Ti cell temperatures are set to give fluxes ~1x10^{13} at/(cm^2-s) using a quartz crystal microbalance (QCM) in the same oxygen conditions as the growth (partial pressure of molecular oxygen P_{O2} or plasma/ozone source), taking into account the oxidation state of the element and tooling factors if known. The starting Sr and Ti shutter times for a nominal Sr_1/Ti_1 cycle are calculated from the QCM fluxes using the substrate's monolayer areal density (e.g. 6.56x10^{14} atoms/cm^2 for STO). Prior to growth, the substrate is annealed at ~700-800°C at the growth pressure for 30m and the RHEED geometry is set, aligning the beam along the (100) azimuth and adjusting the incidence angle to maximize the specular intensity by placing the specular reflection at the intersection of the primary Kikuchi lines, as described in the main text. RHEED image acquisition software (kSA 400, k-Space Associates, Ann Arbor, MI) is set to follow the average intensities within ROIs defined around the diffracted and specular spots. For calibration, a TiO_2-terminated STO substrate is preferred but any substrate with close enough lattice match (LSAT, NGO, DSO…) of sufficiently high surface quality can be used, since the model in Fig. 2 applies independently of the strain state.

As described in Fig. 3a in the main text ("relative Sr:Ti flux calibration"), when starting from a TiO_2-terminated STO substrate, the initial A-site shutter time is set ~10% higher than calculated from the QCM flux estimate (e.g. nominally $Sr_{1.10}/Ti_1$ cycles). Growth is started and after 5-10 cycles a double-peak in the diffracted intensity oscillations will develop; if not, the Sr is increased until they appear. If the substrate starts with AO or mixed termination (often seen on as-received NGO or LAO, or untreated STO), the first oscillations may already show a double-peak or even inverted triangular shape. In this case, the Sr shutter time should be reduced relative to Ti (e.g. nominally $Sr_{0.9}/Ti_1$ cycles) and enough cycles deposited to bring the oscillations to the shallow double-peak shape that is characteristic of cycles starting on the ~0.5 SrO/TiO_2 surface, as shown between 1000-2500s in Fig. 3a. This process will look similar to Fig. 2d, regions [E']→[D']→[C'] but may require more cycles.

Once a shallow double-peak is established, the Sr shutter time per cycle is adjusted until the double-peak shape repeats itself without change for ~5-10 cycles. This is most easily accomplished by monitoring the dip intensity, defined as the intensity minimum at the end of Sr/beginning of Ti deposition: if the dip becomes progressively deeper, then the Sr dose/cycle is too large relative to Ti ("Sr > Ti" in Figure 3) and the Sr shutter time should be reduced. If the dip becomes less deep, then Sr < Ti and the Sr shutter time should be increased. A stable double-peak shape for fixed shutter times indicates stable fluxes and a stoichiometric dose/cycle; these shutter times correspond to the correct relative calibration (Sr:Ti = 1) under alternating-shutter growth for these sources and conditions (see the discussion of source oxidation and differential pumping in Ref. [19], main text). Note that correct relative calibration does not imply each cycle deposits a full monolayer dose, i.e. cycles may be $Sr_{0.9}/Ti_{0.9}$ or $Sr_{1.1}/Ti_{1.1}$. If the dose/cycle is more than ~5-10% away from a full monolayer, interpretation of the double-peak oscillations can be

influenced by the envelope (Refs. [45,47], main text), which disappears after absolute calibration. At this point the Sr and Ti shutter times are not equal because their fluxes are not yet matched, only the doses.

Once the shutter times for the relative calibration are known, “flux-matching” conditions can be determined. Leaving the Ti cell temperature fixed, Sr_1/Ti_1 cycles are continued and the Sr cell temperature is changed and Sr shutter time adjusted to bring both Sr and Ti shutter times equal, maintaining the same double-peak shape used in the relative calibration. For example, in our system under typical STO growth conditions, a Sr cell temperature of ~470°C yields a monolayer shutter time of ~60s at P_{O2} ~ $5x10^{-6}$ torr, and ±1°C change in cell temperature causes about ∓2s change in shutter time.

At this point, the Sr and Ti fluxes are equal (though unknown) and the absolute calibration can now be determined via codeposition. The shutters are opened simultaneously and the period of the specular or diffracted intensity oscillations is measured. Typically >10 oscillations are collected and fit to a damped cosine function (discarding the first few). The oscillation period represents the shutter time, identical for both Sr and Ti, for deposition of a full $Sr_{1.00}Ti_{1.00}O_3$ unit cell that determines the absolute calibration of the two fluxes in atoms/(cm^2-s). If desired, the growth conditions ($T_{substrate}$, P_{O2}) can be changed and the procedure for relative and absolute calibration can be repeated, to optimize the conditions (see below).

After the flux calibration is completed, a new substrate is transferred into the MBE and the desired film/heterostructure can be grown by shutter and/or codeposition methods. If the shutter method is used for growth, any flux variations will be seen in small changes of the double-peak shape (the dip becoming deeper or shallower) and the shutter times can be adjusted slightly to compensate and maintain a stable shape. At any point, the surface termination can be set, i.e. TiO_2 by deposition of the necessary amount of Ti as determined from the oscillations using the model in Fig. 2 of the main text.

Other A- and B-site fluxes can be calibrated based on the STO calibration, as shown in Supplementary Figure 3 and described next. The STO calibration serves as reference for the mixed-A-site calibrations $(Sr_{0.5}Eu_{0.5})TiO_3$, $(Sr_{0.5}La_{0.5})TiO_3$, $(Sr_{0.5}Nd_{0.5})TiO_3$ that are then used to grow the high-entropy oxide $(Sr_{0.25}Eu_{0.25}La_{0.25}Nd_{0.25})TiO_3$. Mixed-B-site calibrations can be performed following the same procedure as described for mixed A-site.

Here we extend the calibration method of Figure 3 in the main text to permit the growth of more complex phases. Generally, for each multielemental phase that is targeted, a growth plan must be defined that is specific to that particular phase. The complexity of the growth plan will depend on the desired final phase. For example, a final target of $Sr_{1-x}Eu_xTiO_3$ with x = 0.5 will have a growth plan with fewer constraints on the fluxes than the HEO $(Sr_{0.25}Eu_{0.25}La_{0.25}Nd_{0.25})TiO_3$.

Before starting the calibration procedure for $(Sr_{0.25}Eu_{0.25}La_{0.25}Nd_{0.25})TiO_3$ shown in Supplementary Figure 3, we assume the Sr and Ti fluxes for STO have been matched and the absolute calibration has been completed, so that the shutter time is known that gives a monolayer dose of Sr or Ti. The growth plan for $(Sr_{0.25}Eu_{0.25}La_{0.25}Nd_{0.25})TiO_3$ requires first the calibration of Eu, La and Nd fluxes relative to Sr. This is accomplished by using the STO calibration to grow $Sr_{0.5}Eu_{0.5}TiO_3$, $Sr_{0.5}La_{0.5}TiO_3$ and $Sr_{0.5}Nd_{0.5}TiO_3$ in such a way as to match the Eu, La and Nd fluxes with Sr, for optimized growth of $(Sr_{0.25}Eu_{0.25}La_{0.25}Nd_{0.25})TiO_3$.

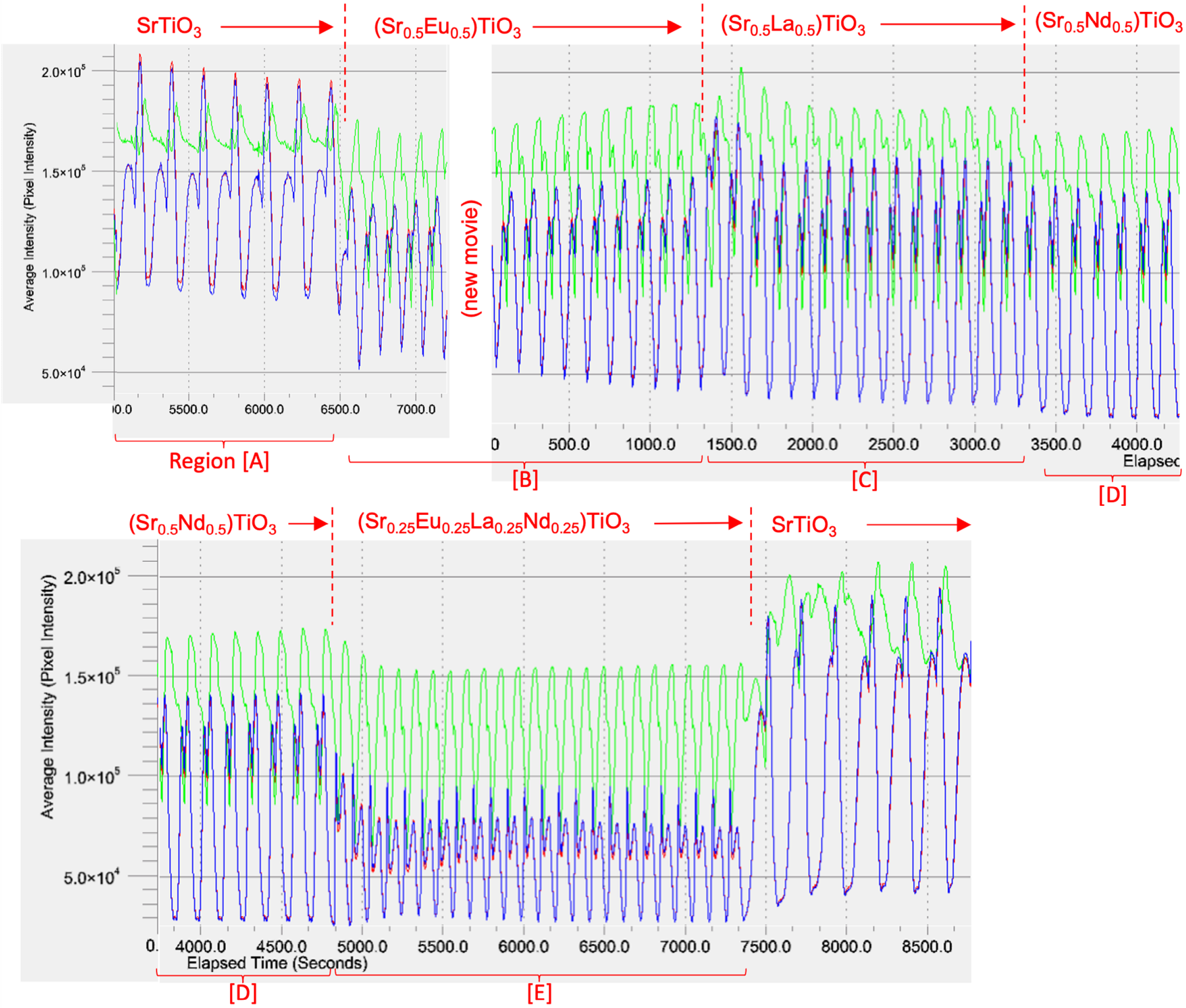

Supplementary Fig. 3 – RHEED diffracted-intensity "double peak" oscillations during flux calibration of $(Sr_{0.25}Eu_{0.25}La_{0.25}Nd_{0.25})TiO_3$. Starting from ~0.5 partial SrO coverage on TiO_2 ([region A]) and calibrated Sr_1/Ti_1 cycles, a sequence of calibration layers are grown for $(Sr_{0.5}Eu_{0.5})TiO_3$ ([B], using $(Sr+Eu)_1/Ti_1$ cycles), $(Sr_{0.5}La_{0.5})TiO_3$ ([C]) and $(Sr_{0.5}Nd_{0.5})TiO_3$ ([D]) before $(Sr_{0.25}Eu_{0.25}La_{0.25}Nd_{0.25})TiO_3$ ([E]) is grown. Note that stable, repeatable double-peak oscillations are seen in all regions, indicating that correct stoichiometry is obtained for all layers. Note also that the A-site shutter time is twice the Ti shutter time in region [A], equal in regions [B], [C] and [D], and half in region [E], as described in the growth plan for the HEO oxide. Diffracted intensities are red and blue (their equal amplitudes indicate proper beam alignment along the (100) azimuth); specular is green. Growth conditions: T_{subs} = 750°C, $P_{O2} = 1x10^{-7}$ torr.

We start with $Sr_{0.5}Eu_{0.5}TiO_3$ calibration. The goal is to have equal Sr and Eu shutter times, implying for this stoichiometry that their fluxes must be matched. According to the method described in Figure 3 of the main text, stable double-peak oscillations indicate a stoichiometric ratio of (A + A'):B = 1 (here A = Sr, A' = Eu). In general, when growing a phase like $Sr_{1-x}Eu_xTiO_3$ for some x, the Sr and Eu fluxes should be adjusted so that their shutter times are equal during the A-site portion of the cycle; growth is usually

improved by simultaneous arrival of all A- or B-site elements to give correct instantaneous stoichiometry. For $x = 0.5$ this requires that Sr and Eu fluxes be matched. (The general case for $x \neq 0.5$ is discussed below). Furthermore, codeposition of $Sr_{0.5}Eu_{0.5}TiO_3$ requires that the (Sr + Eu) shutter time be equal to the Ti shutter time; this constrains the Sr and Eu fluxes to be half of the Ti flux ($F_{Sr} = F_{Eu} = ½ F_{Ti}$). The utility of setting fluxes in this ratio to allow codeposition of $Sr_{0.5}Eu_{0.5}TiO_3$ will become apparent below.

Here we implement this plan for $Sr_{0.5}Eu_{0.5}TiO_3$ growth. After the STO calibration is completed, shuttered growth of STO is continued, keeping the double-peak shape constant while the Sr cell temperature is further lowered (leaving the Ti cell temperature fixed) until the Sr shutter time becomes twice the Ti shutter time ($F_{Sr} = ½ F_{Ti}$). In Supplementary Figure 3, this twice-longer Sr shutter time compared to Ti is visible in the double-peak oscillation shape in region [A]. To calibrate $Sr_{0.5}Eu_{0.5}TiO_3$, the original QCM measurements were done to set the Eu cell temperature such that the Eu flux is roughly half the Ti flux; thus, at this point the Sr and Eu fluxes will be approximately matched to each other. Starting at time ~6500s of Supplementary Figure 3, the Sr and Eu shutter times are set to half of their full-layer time and $(Sr_{0.5} + Eu_{0.5})_1/Ti_1$ cycles are begun, opening the (Sr + Eu) shutters together during the A-site portion of the cycle. While depositing, the (Sr + Eu) shutter time is adjusted until the double-peak oscillations repeat stably. Then the Eu cell temperature is adjusted (up or down) together with the (Sr + Eu) shutter time (down or up) until eventually the (Sr + Eu) shutter time again becomes equal to the Ti shutter time. Proper flux-matching is confirmed by maintaining a stable double-peak shape for ≥5 cycles for fixed shutter times. Once accomplished, $F_{Sr} = F_{Eu} = ½ F_{Ti}$ and each $(Sr_{0.5} + Eu_{0.5})_1/Ti_1$ cycle will yield one full unit cell of $Sr_{0.5}Eu_{0.5}TiO_3$ with equal A- and B-site shutter times. Codeposition can be done to check the oscillation period; it should be identical to the STO codeposition period determined originally, confirming that the Ti flux has not changed.

In Supplementary Figure 3, from the previous day's calibration the Eu cell temperature was already close to that needed to flux-match Eu to Sr for $x = 0.5$, and stable double-peak oscillations are achieved quickly; only 15 unit cells of $(Sr_{0.5}Eu_{0.5})TiO_3$ are required to confirm stoichiometric and flux-matched growth.

To grow the HEO phase $(Sr_{0.25}Eu_{0.25}La_{0.25}Nd_{0.25})TiO_3$, each of the four A-site fluxes (Sr, Eu, La, Nd) needs to be matched to half the Ti flux. For this, the above procedure for $(Sr_{0.5}Eu_{0.5})TiO_3$ is repeated for $(Sr_{0.5}La_{0.5})TiO_3$ (region [C] in Supplementary Figure 3) and $(Sr_{0.5}Nd_{0.5})TiO_3$ (region [D]). Growth of $(Sr_{0.25}Eu_{0.25}La_{0.25}Nd_{0.25})TiO_3$ phase is now possible, since the common A-site shutter time for ($Sr_{0.25}$ + $Eu_{0.25}$ + $La_{0.25}$ + $Nd_{0.25}$) deposition will be equal to one-quarter of the Sr full-layer shutter time (= half the Ti shutter time). This difference in A- and B-site shutter times per cycle can be seen during the HEO growth (region [E]). Slight adjustment of the (common) A-site shutter time may be needed to maintain repeatable double-peak oscillations, but which of the four A-site fluxes has drifted cannot be known without returning to growth of the individual $ATiO_3$ phases until the culprit A-site flux is indentified. We find the Ti flux is the most stable of the elements in the HEO phase and so it is assumed constant throughout the calibration procedure; any drift will show up in a change of the codeposition oscillation period.

The XRD symmetric 2θ-ω scan of an HEO $(Sr_{0.25}Eu_{0.25}La_{0.25}Nd_{0.25})TiO_3$ film grown using the above calibration procedure is shown in Supplementary Fig. 4.

The calibration procedure of $Sr_{1-x}Eu_xTiO_3$ for $x \neq 0.5$ can be generalized from the previous $x = 0.5$ case; for brevity we ignore the requirement of matching (Sr + Eu) and Ti fluxes as described above (we assume we do not want to codeposit the $Sr_{1-x}Eu_xTiO_3$ phase we are preparing, though of course that can be done with an additional step, if desired). The goal is to adjust the Sr and Eu fluxes so that their shutter times are equal during the A-site portion of the cycle and their flux ratio is correct for a given x. Only for the special case of $x = 0.5$ will the Sr and Eu fluxes be matched; for all other x, the Eu flux must be a factor $x/(1 - x)$ of the Sr flux. The previous approach can be directly applied, and details will be described in a separate study as noted below. Note that for very low doping ($x < 0.05$ or > 0.95), matching between A-site fluxes may not be practical time-wise, and some compromise should be decided for their shutter times, i.e. the two A-site shutter times may be different during the A-site part of the cycle.

The ability to grow precisely stoichiometric $Sr_{1-x}Eu_xTiO_3$ films (or similarly $La_{1-x}Sr_xMnO_3$, $La_{1-x}Sr_xFeO_3$ and others) for any x allows the construction of the phase diagram for $0 < x < 1$. Some general considerations are worth mentioning. Eu^{2+} can be substituted for Sr^{2+} for any value of x, with nearly constant lattice parameter (both STO and ETO are cubic with a = 3.905 Å). Care must be taken to determine a P_{O2} compatible with a given x, since the end-phases STO and ETO have quite different optimized P_{O2}. While cation-stoichiometric STO can be grown by the shutter method at any pressure between $5x10^{-9} < P_{O2} < 2x10^{-5}$ torr following the procedure outlined in Figure 3 of the main text, STO will be oxygen deficient for P_{O2} less than ~$8x10^{-7}$ torr in our system. In contrast, pure perovskite-phase ETO, like $LaTiO_3$ and $NdTiO_3$, only grows well in a small window near $P_{O2} \sim 3x10^{-8}$ torr (Ref. [61], main text). The optimal P_{O2} window for ETO can be determined by following the calibration procedure outlined above in different P_{O2}: within the optimized window, the RHEED intensity and amplitude of the double-peak oscillations are constant; outside the window, the intensity and oscillation amplitude decrease and the diffuse background increases over repeated cycles. Consequently, for a mixed phase like $Sr_{1-x}Eu_xTiO_3$, the optimal PO_2 will depend on x: for large x, a P_{O2} ~$3x10^{-8}$ torr is required, while for small x, $P_{O2} > 1x10^{-6}$ torr is possible. For $x = 0.5$, we find that an intermediate pressure P_{O2} ~$1x10^{-7}$ torr works well for all three phases $Sr_{0.5}Eu_{0.5}TiO_3$, $Sr_{0.5}La_{0.5}TiO_3$ and $Sr_{0.5}Nd_{0.5}TiO_3$. In general, this "compromise" pressure depends on the parent phases and x, and must be determined via growth experiments using the procedures outlined above.

Substrate temperature can be varied by several hundred degrees and oxygen pressure by several orders of magnitude during optimization using similar criteria as mentioned above; once the film surface shows signs of disorder or 3-D islands, there are two options: either 1) the surface can be recovered by depositing 10-20 layers of stoichiometric STO (whose growth windows in P_{O2} and $T_{substrate}$ are quite large), or 2) a new calibration substrate can be transferred and calibration continued. In this way, optimization of a new phase can typically be performed on one or two substrates; mapping an entire phase diagram takes correspondingly more time but can be done entirely *in situ*, and can be confirmed by *ex situ* XRD once optimization is completed. Calibration of a second B-site flux can be performed in the same way as described above for mixed A-site calibration, by substituting for Ti and maintaining a stable double-peak shape. Specific descriptions applying this approach to the growth optimization of different mixed-valent perovskite families will be reported separately.

A few concluding remarks can be made. The relative calibration method presented here has advantages when performed with the "shallow double-peak" shape compared to a triangular shape. For STO, this

corresponds to a starting surface with ~0.5 layer SrO coverage on TiO_2, instead of the "triangular" shape that corresponds to <0.3 layer SrO on TiO_2. First, in the shallow double-peak regime the RHEED intensity at the dip between peaks is most sensitive to small changes in the partial A-site coverage because of the steep slope when ending A-site and starting B-site deposition; thus, the dip intensity level can be more precisely tracked and maintained constant. Second, when the growth conditions are not optimal and the overall RHEED intensity is gradually decreasing, the oscillation shape will still retain the same double-peak shape if A:B = 1 even though the overall intensity has a decaying envelope (i.e. the relative calibration can be performed outside of the optimal growth window). As a consequence, the double-peak regime has two parameters that can be tracked separately (ratio of dip intensity to starting/ending intensity, and overall intensity) whereas the triangular regime has only one parameter (the overall intensity). For these reasons, the double-peak regime (Fig. 2d, regions [C] and [C']) is preferred over triangular (Fig. 2d, [A] and [B], [A'] and [B']).

As discussed in the main text, we find experimentally that the double-peak method applies equally well for both nonpolar and polar perovskites for (001) growth. Nonetheless, we consistently observe differences between polar and nonpolar RHEED oscillations: the overall RHEED intensity is typically reduced for polar growth; excess A-site should be kept below ~0.3 layers on the BO_2 surface before the beginning of a cycle; and the inflection point in the A-site part of the cycle is typically closer to 1.0 AO layers on the BO_2 surface. A starting surface with too much excess A-site (i.e. having a dip intensity that is too deep, for example as in Figure 2d regions [D], [E] and [D']) usually leads to surface roughening that cannot be recovered subsequently even with STO. Because of these tendencies, it is most time-efficient to start with nonpolar calibration and follow with the substitutional method for 3^+-valence elements, as they produce equivalent results when care is taken.

Since this calibration method yields absolute fluxes, Ruddlesden-Popper phases can be grown by controlling the shutter times, e.g. Sr_2/Ti_1 cycles for Sr_2TiO_4 (n =1), and combinations of STO and Sr_2TiO_4 unit cells for higher *n*. By appropriate flux matching, codeposition of spinel and other phases are also possible and can be optimized over the same windows of P_{O2} and $T_{substrate}$ in which the corresponding perovskite phases are stable.

Finally, we note that the calibration method described here will establish quite precise stoichiometry and dose only at the position on the substrate where the RHEED beam is focused (usually the center). Effusion cells are typically aligned away from the substrate normal by 20-40° in most MBE chambers, causing stoichiometry gradients of a few percent across a 10x10 mm^2 area (see for example M. Warusawithana et al, Nat. Commun. **4**, 2351 (2013)). The double-peak calibration method presented here can easily detect such gradients on different points of the substrate during growth, e.g. for 2% difference in A- and B-site fluxes at opposite edges of the substrate, after depositing 50uc the two edges should have opposite terminations. These stoichiometry gradients can be removed by continual azimuthal rotation (CAR) during growth. The shuttered growth method to control stoichiometry can be implemented during rotation by synchronizing the sampling of RHEED images with the CAR rotation to follow the double-peak oscillations, as done in semiconductor growth. Growth of heterostructures and superlattices will require synchronizing the rotation frequency with the fluxes, to ensure the deposition of a unit cell of material is completed in an integral number of rotations and can be tracked by the oscillations.

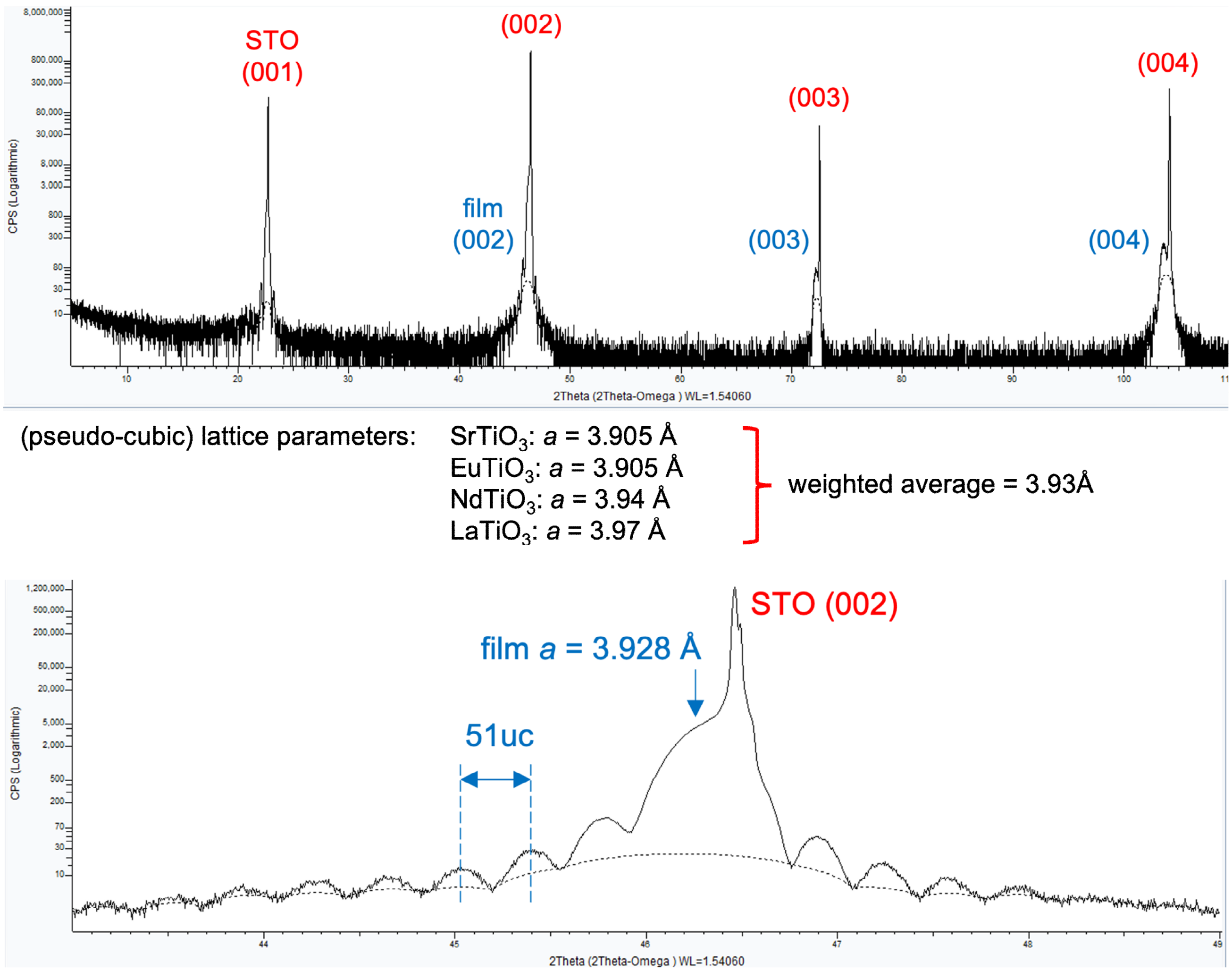

Supplementary Figure 4 – XRD on 50uc ($(Sr_{0.25}Eu_{0.25}La_{0.25}Nd_{0.25})TiO_3$) film on STO.
High-resolution wide-range 2θ-ω scan of HEO film grown on STO(001) substrate. Only the expected (00L) film peaks are seen, and the film c-axis lattice parameter is close to the weighted average of the parent phases. Finite-thickness fringes confirm the desired film thickness planned from the calibration.

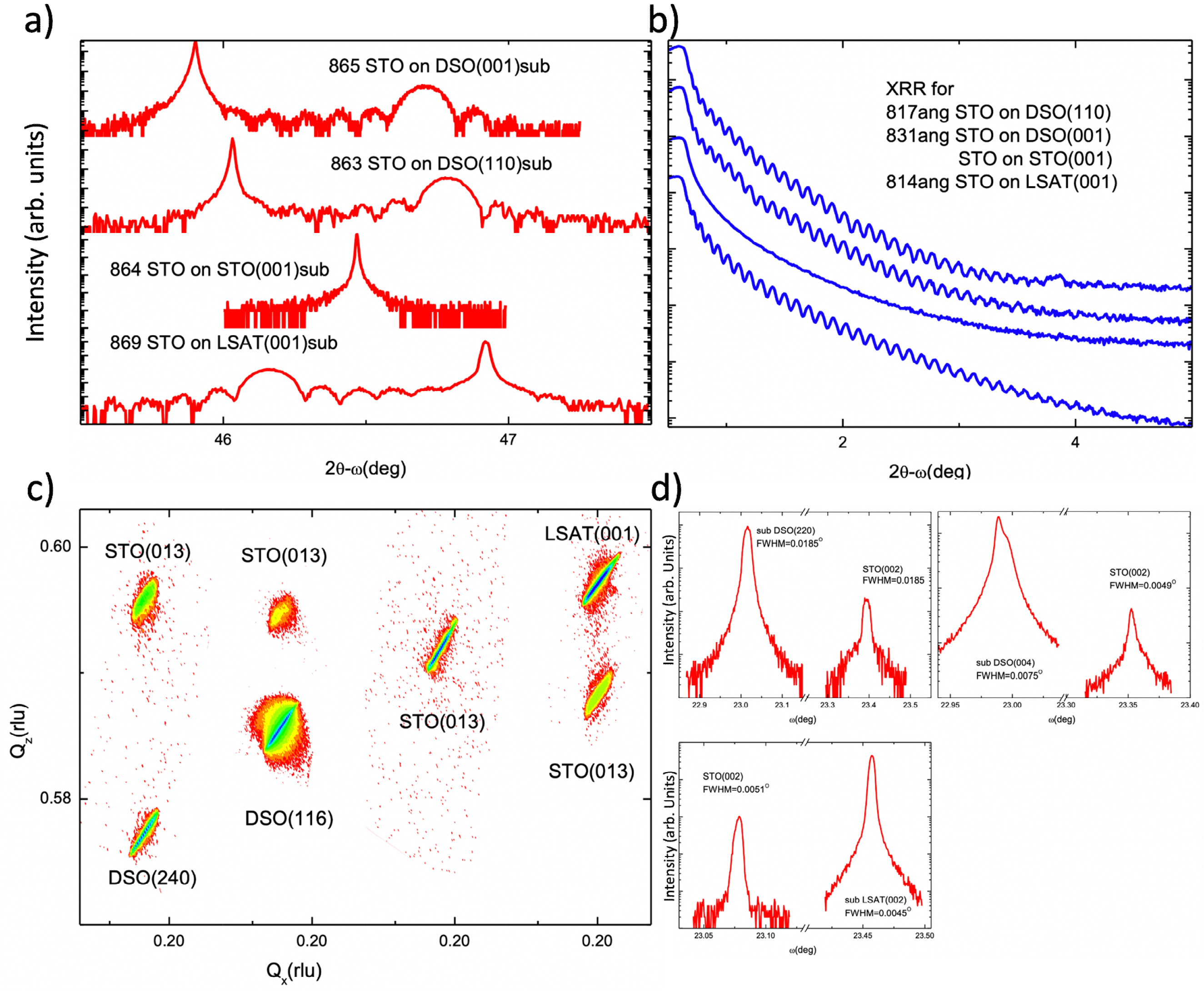

Supplementary Fig. 5 – XRD on STO films grown on different substrates by shutter method.

(a) High-resolution 2θ-ω scans near the (002) reflection of STO films grown on LSAT(001), STO(001), DSO(110) and DSO(001) substrates, using 1.5406 Å (Cu $K_{\alpha 1}$) radiation. Finite-thickness fringes are seen on all except for the STO substrate. Scans are shifted for clarity.

(b) X-ray reflectivity scans for films in (a), showing thickness oscillations on all except the STO substrate. Scans are shifted for clarity. Thicknesses from fits are shown.

(c) Reciprocal space maps (RSMs) for films in (a), showing the STO films are coherently strained on all substrates.

(d) Rocking curves for films in (a). FWHM values for films are the same or slightly narrower than the corresponding substrate, confirming the high-quality of the grown films.

(e) Growth conditions: 730°C (optical pyrometer), PO_2=4x10^{-6} mBar.

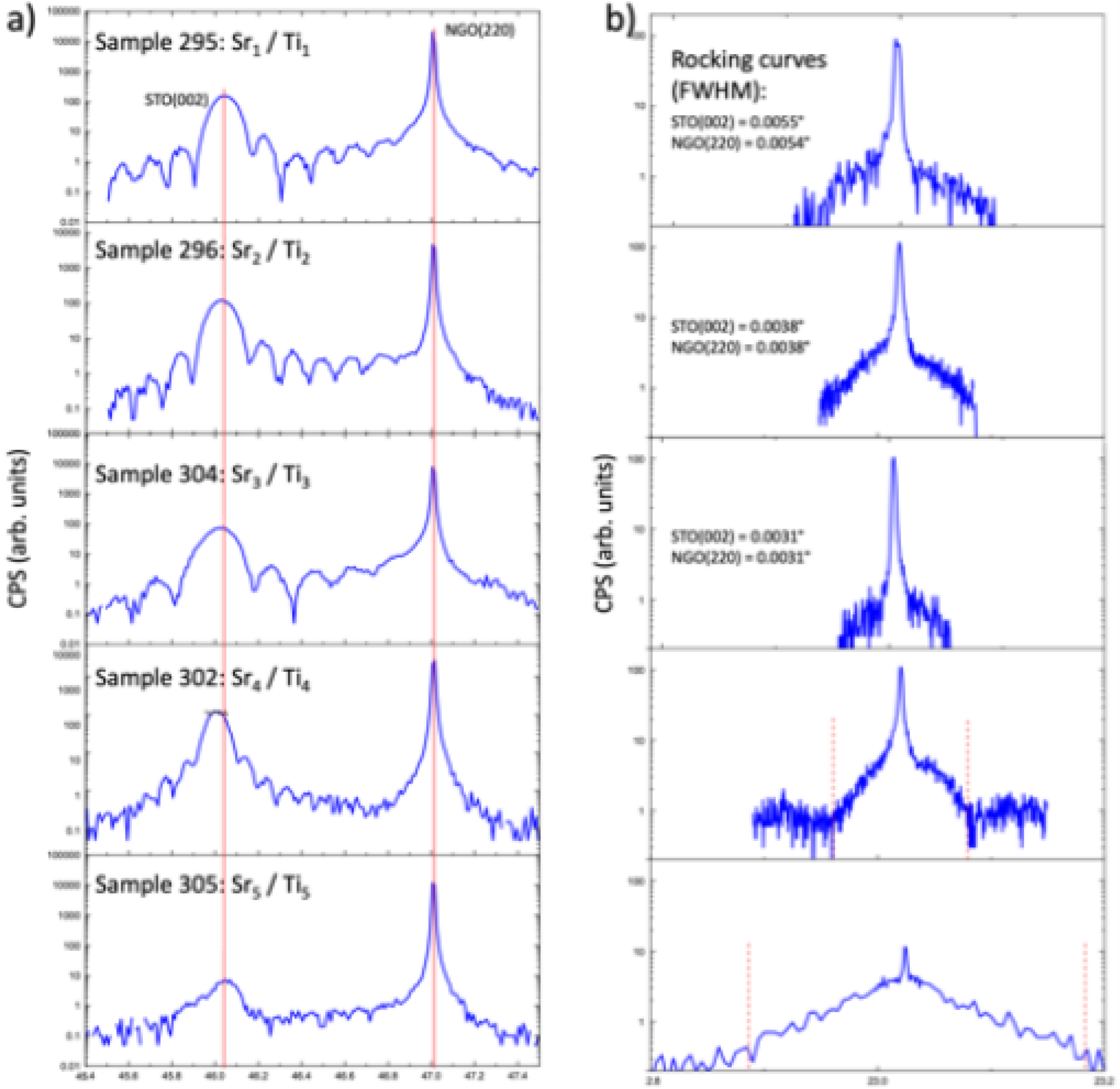

Supplementary Figure 6 – XRD on STO films grown with Sr_n / Ti_n cycles, $1 \leq n \leq 5$.

a) High-resolution 2θ-ω scans near NGO(220) reflection for STO films grown on NGO(110).

b) Rocking curves of STO films in (a). Note for $n \geq 4$, a broad background appears underneath the main peak.

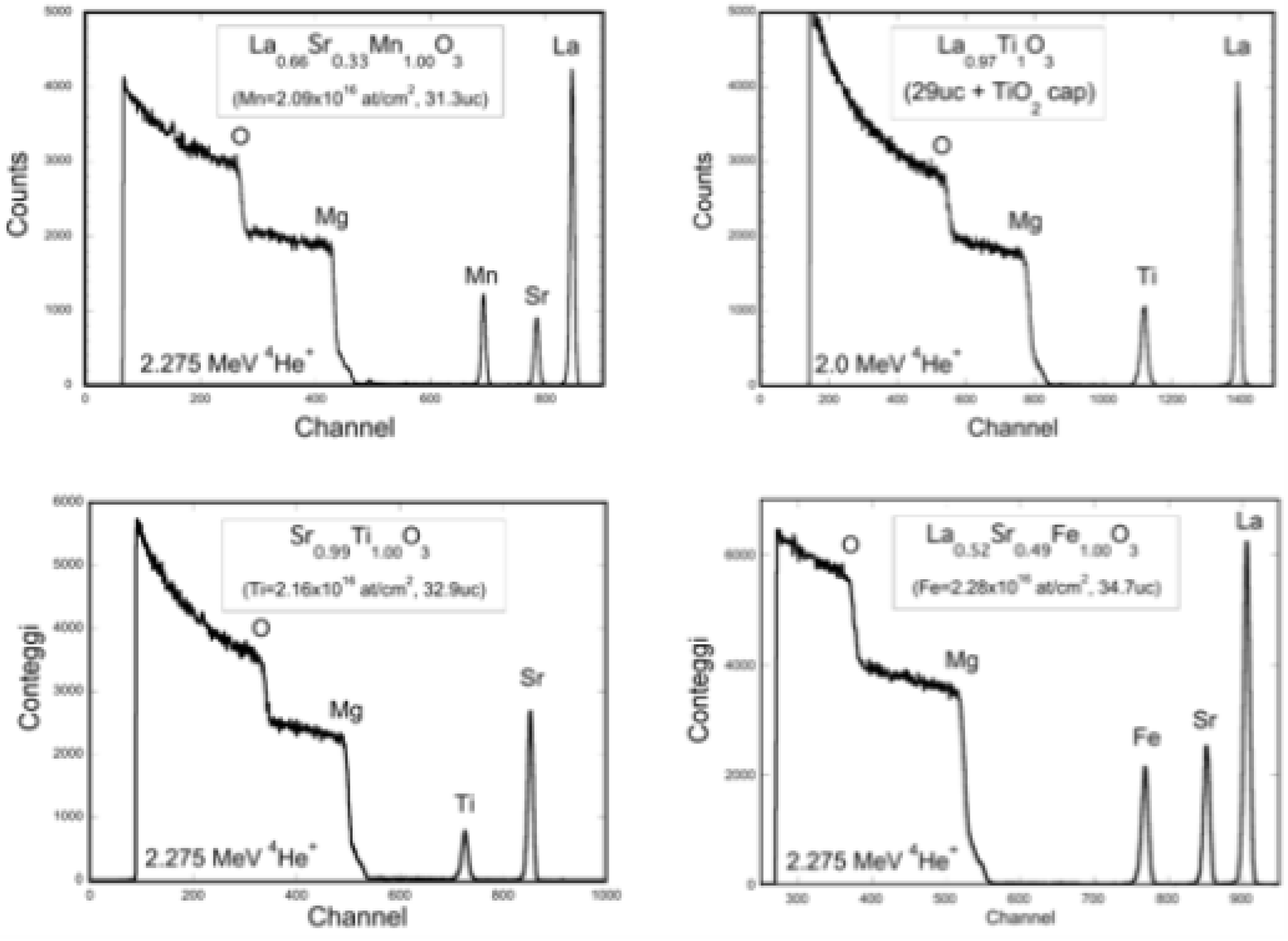

Supplementary Figure 7 – Rutherford backscattering spectrometry (RBS) spectra for different perovskite films. Films ~30uc thick on MgO(001) substrates grown simultaneously with adjacent STO substrates on which the shutter method is used to control stoichiometry. Measured compositions are close to desired stoichiometry, within the error bars of the measurements (± 3%). Target stoichiometries:

a) $La_{2/3}Sr_{1/3}MnO_3$
b) $LaTiO_3$
c) $SrTiO_3$
d) $La_{1/2}Sr_{1/2}FeO_3$.

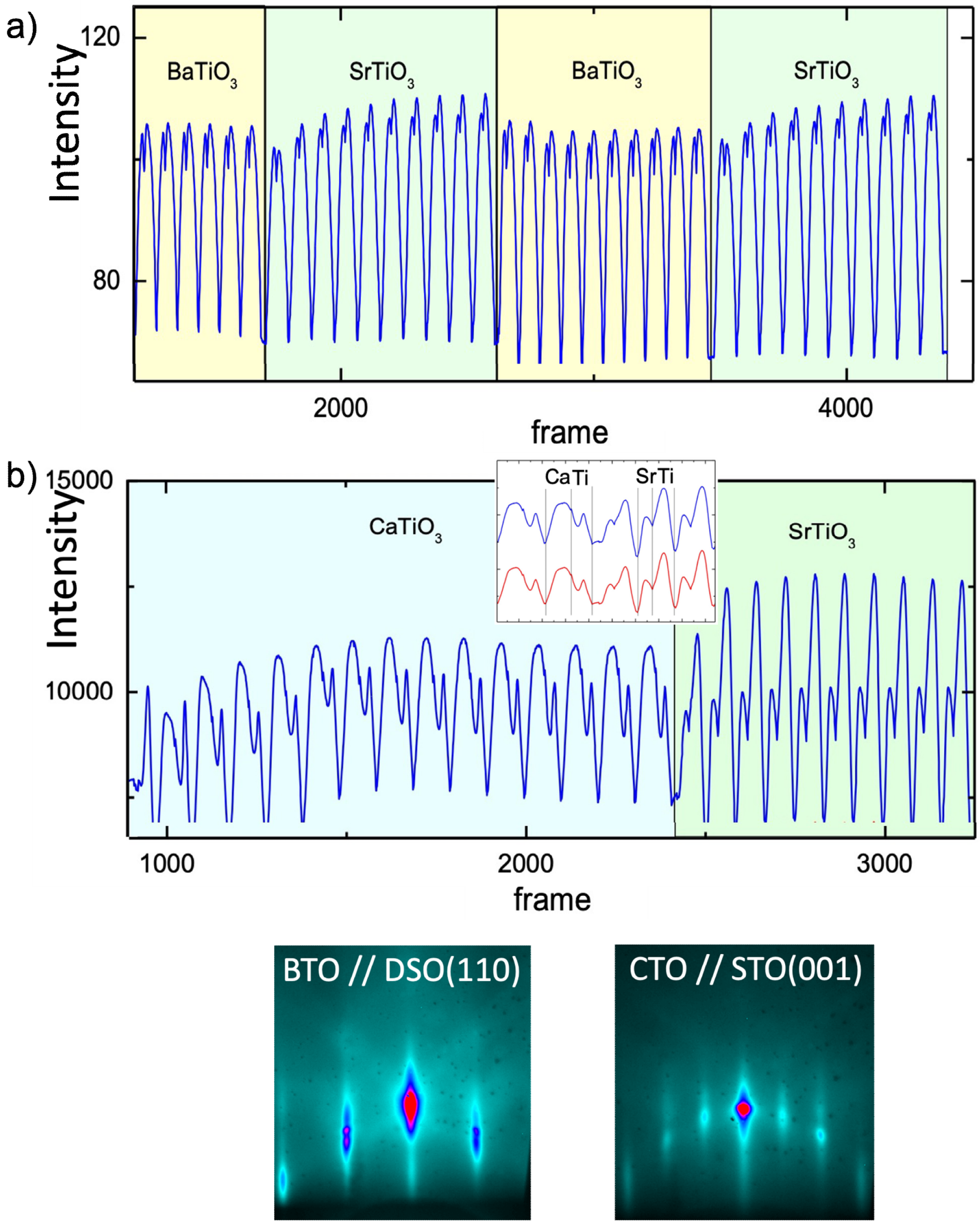

Supplementary Figure 8 – Titanate superlattices by shuttered growth (MBE).

a) STO-BTO//DSO(110), growth conditions: P_{O2}=5x10^{-6} mBar, $T_{substrate}$=730°C.

b) STO-CTO//NGO(110), same growth conditions as (a).

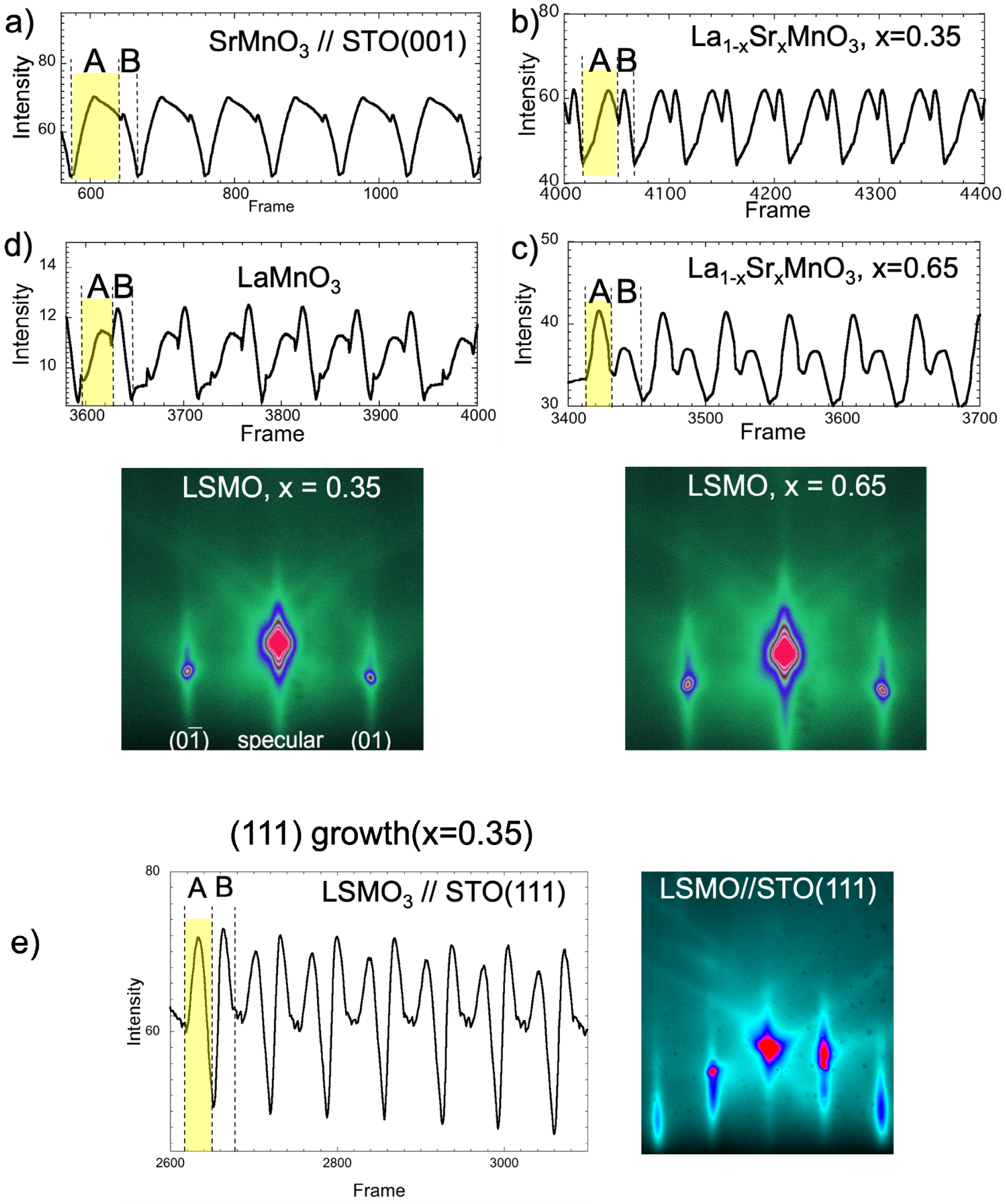

Supplementary Fig. 9 – Manganite phases on STO by shuttered growth (MBE).

a) SMO//STO(001), growth conditions: P_{O2}=5x10^{-6} mBar, $T_{substrate}$=800°C

b) LSMO (x=0.35)//STO(001), growth conditions: P_{O2}=2x10^{-6} mBar, $T_{substrate}$=760°C

c) LSMO (x=0.65)//STO(001), growth conditions: P_{O2}=5x10^{-7} mBar, $T_{substrate}$=775°C

d) LMO//STO(001), growth conditions: P_{O2}=1x10^{-6} mBar, $T_{substrate}$=760°C

e) LSMO (x=0.35)//STO(111), growth conditions: P_{O2}=2x10^{-6} mBar, $T_{substrate}$=740°C

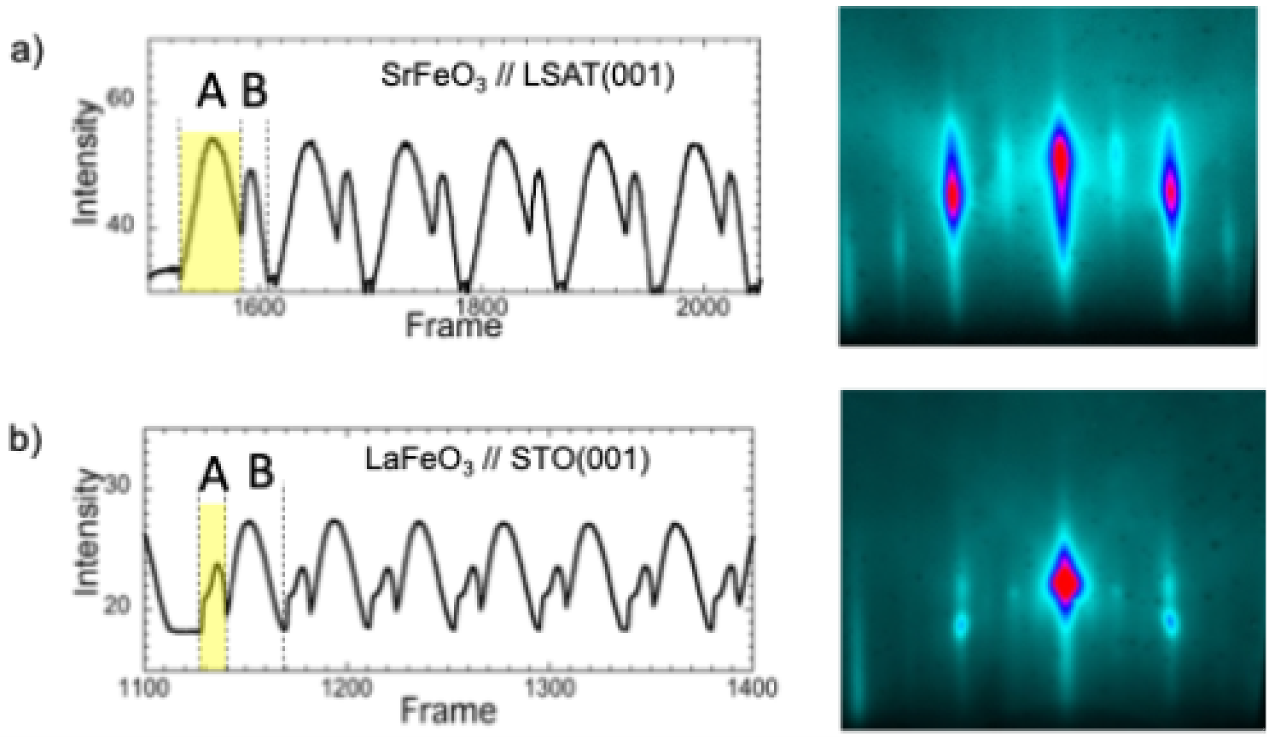

Supplementary Figure 10 – Ferrite phases on STO by shuttered growth (MBE).

a) $SrFeO_3$//LSAT(001), growth conditions: P_{O2}=5x10^{-6} mBar, $T_{substrate}$=750°C

b) $LaFeO_3$//STO(001), growth conditions: P_{O2}=1x10^{-6} mBar, $T_{substrate}$=730°C

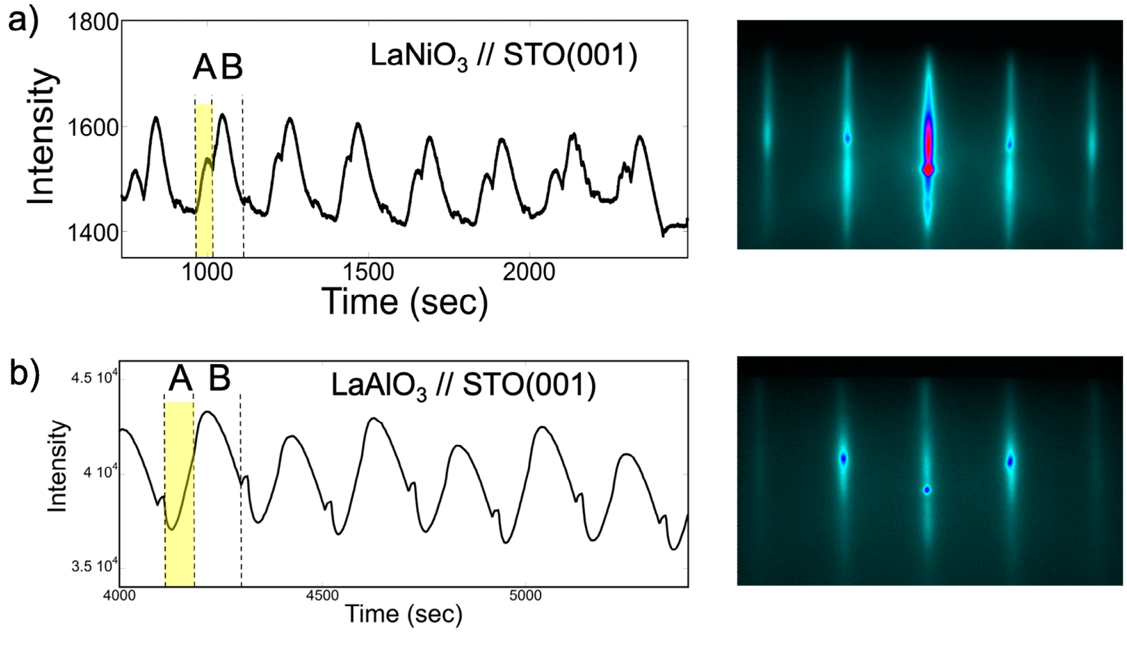

Supplementary Fig. 11 – $LaNiO_3$ and $LaAlO_3$ on STO(001) by shuttered growth (MBE).

c) LNO//STO(001), growth conditions: P_{OZONE}=2x10^{-5} mBar, $T_{substrate}$=580°C

d) LAO//STO(001), growth conditions: P_{OZONE}=2x10^{-5} mBar, $T_{substrate}$=680°C

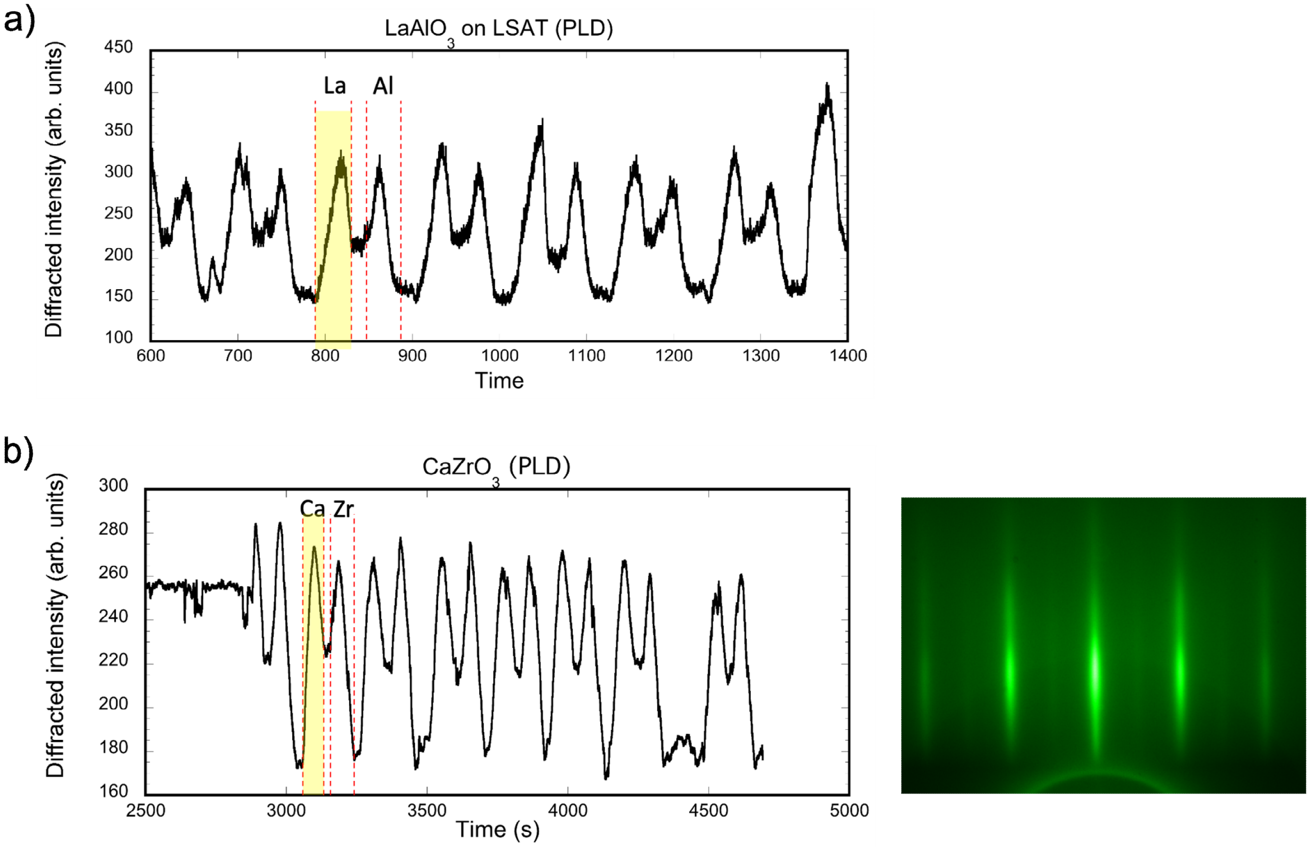

Supplementary Fig. 12 – Pulsed-laser deposition (PLD) of $LaAlO_3$ and $CaZrO_3$ using sequential ablation of binary oxide targets, showing "double-peak" oscillations during growth. Note the waiting time between A- and B-site layer that is needed to allow rotation of target carousel to select the desired oxide targets: La_2O_3 and Al_2O_3 for $LaAlO_3$; CaO and ZrO_2 for $CaZrO_3$.

a) $LaAlO_3$//LSAT(001), growth conditions: P_{O2}=50 mTorr, $T_{substrate}$=650°C
b) $CaZrO_3$//STO(001), growth conditions: P_{O2}=10 mTorr, $T_{substrate}$=700°C

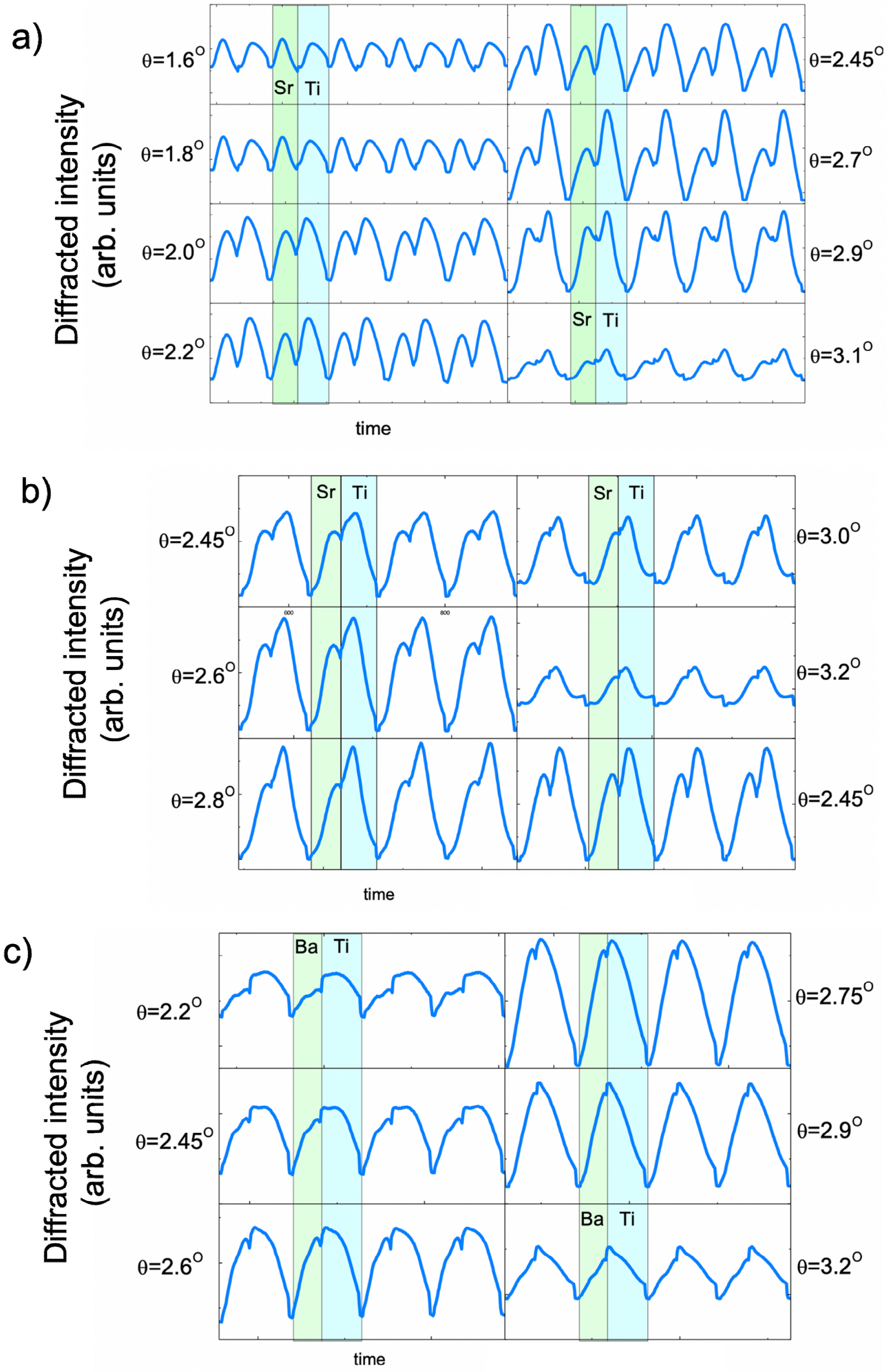

Supplementary Fig. 13 RHEED diffracted (10) intensity oscillations along (100) azimuth during stoichiometric growth at different incidence angles θ. Excess A-site ~ 0.5 layers on TiO_2 in each, and scale is similar for all graphs in each (a), (b) and (c).

a) STO growth on STO(001) substrate at different incidence angles: between incidence angles θ = 2-2.8°, the characteristic double-peak shape described in Fig. 2 (main text) is present and can be used for stoichiometry calibration.
b) STO growth on DSO(110) substrate at different incidence angles.
c) $BaTiO_3$ growth on DSO(110) substrate at different incidence angles.

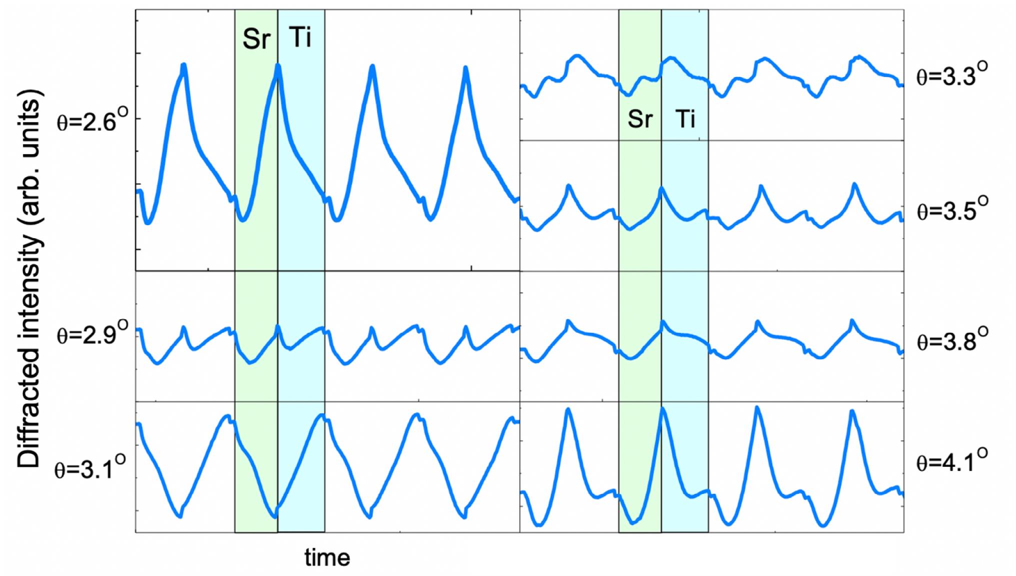

Supplementary Fig. 14 RHEED diffracted (11) intensity oscillations during stoichiometric $SrTiO_3$ growth on LSAT(001) substrate at different incidence angles. Note that in a narrow range of incidence angle $\theta = 2.6\text{-}3.1°$, all three double-peak shapes are seen: upward triangular, frequency-doubled, and downward triangular. Scale is same for all graphs.
